# Risk factors for cannabis use disorders and cannabis psychosis in Japan: Second report of a survey on cannabis‐related health problems among community cannabis users using social networking services

**DOI:** 10.1002/npr2.12307

**Published:** 2022-12-19

**Authors:** Yuji Masataka, Takeshi Sugiyama, Yoshiyuki Akahoshi, Toshihiko Matsumoto

**Affiliations:** ^1^ General Incorporated Association Green Zone Japan Saitama Japan; ^2^ Department of Neurology Kumamoto Seijo Hospital Kumamoto Japan; ^3^ College of Bioresource Sciences Nihon University Fujisawa Kanagawa Japan; ^4^ Department of Drug Dependence Research, National Institute of Mental Health National Center of Neurology and Psychiatry Tokyo Japan

**Keywords:** cannabis, Japan, marijuana abuse, mental health, psychotic disorders

## Abstract

**Aim:**

To determine the risk factors for cannabis use disorders and cannabis psychosis in Japan based on a 2021 online survey among Japanese users of social network services.

**Methods:**

The 3142 respondents who had used cannabis within the preceding year were categorized into two groups based on the development of cannabis use disorder and/or cannabis psychosis. Analyses were performed to determine these conditions' risk factors.

**Results:**

Multivariate analysis revealed that factors significantly associated with cannabis use disorders were “cannabis‐use initiation at a young age” (*p* = 0.004, OR: 0.951, 95% CI [0.920–0.984]), “family history of mental health problems” (*p* < 0.001, OR: 1.988, 95% CI [1.545–2.556]), “psychiatric disorders preceding cannabis use” (*p* = 0.039, OR: 1.672, 95% CI [1.026–2.722]), and “use of cannabis products other than dry flower” (*p* < 0.001, OR: 2.725, 95% CI [1.844–4.026]). Factors significantly associated with cannabis psychosis were “cannabis‐use initiation at a young age” (*p* = 0.011, OR: 0.888, 95% CI [0.811–0.973]) and “family history of mental health problems” (*p* = 0.002, OR: 2.531, 95% CI [1.400–4. 576]).

**Conclusion:**

Risk factors for problematic cannabis use were cannabis initiation at a young age, pre‐cannabis psychiatric disorders, family history of mental health problems, and the use of cannabis products other than dry flower. However, the causal relationship among these factors remains ambivalent.

## Abstract 2

We examined the risk factors of Japanese cannabis users (*N* = 3142) for cannabis use disorder and cannabis psychosis. We assessed by dividing the user groups by whether or not the user had cannabis use disorder and whether or not the user had cannabis psychosis. Results with statistically significant correlations with cannabis use disorders were “cannabis‐use initiation at a young age,” “family history of mental health problems,” “psychiatric disorders preceding cannabis use,” and “use of cannabis products other than dry flowers.” Results with statistically significant correlations with cannabis psychosis were “cannabis‐use initiation at a young age” and “family history of mental health problems.”

## Graphical abstract

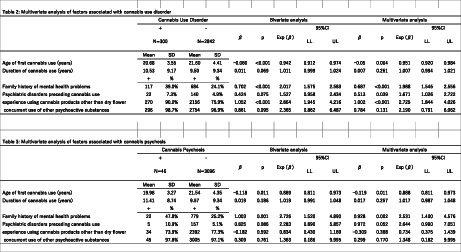

## 1 INTRODUCTION

Cannabis regulations have undergone significant alterations internationally in recent years, with Uruguay emerging as the first country in the world to legalize cannabis for recreational use in 2013, followed by similar decisions by Canada in 2018, Malta in December 2021, and Thailand in June 2022.^1^, ^2^, ^3^

In the United States (US), the federal government generally does not condone cannabis use; however, it does permit cannabis use in Alaska, California, Oregon, Washington, Nevada, Colorado, Michigan, Illinois, Massachusetts, Maine, Vermont, Arizona, Montana, New Jersey, New York, Rhode Island, and New Mexico. Connecticut, South Dakota, Washington DC, and Guam have legalized the recreational use of cannabis through state law.^4^ In addition, Germany, Italy, and Luxembourg have been considering the legalization of cannabis as of June 2022.^5^, ^6^, ^7^ Medical cannabis is an inclusive concept that encompasses cannabis plants, extracts, and medicines containing cannabis ingredients used to alleviate illnesses and symptoms.

An increasing number of areas have permitted legal access to medical cannabis, with approximately 20 countries and 37 US states allowing its use as of June 2022.^4^, ^8^ Based on these countries' decisions, the United Nation Commission on Narcotics reviewed the schedule for cannabis and approved its medical significance in November 2020.^9^

In Japan, the Ministry of Health, Labour and Welfare announced its intention to approve medical cannabis.^10^ This will allow for clinical trials and insurance approval of cannabidiol (CBD) (Epidiolex®️) for the treatment of intractable epilepsy and will allow future research on drugs containing tetrahydrocannabinol (THC) to be treated like narcotics.^11^ Notwithstanding, the government also introduced a policy that maintains the regulation of cannabis as a drug of abuse, and new penalties for cannabis use are being considered under the guise of curbing the increase in cannabis users.^12^ At an experts' meeting, where the revision of the law was deliberated, the dangers of cannabis use were discussed on the basis of toxicity data from animal experiments, and most participants supported the reinforcement of current penalties.^13^

However, on a global scale, the prevailing stance is that the imposition of penalties on drug users, not just cannabis users, should be reconsidered. In 2021, the United Nations Office on Drugs and Crime issued a position statement asserting that drug problems should be addressed by means other than punishment.^14^ From the perspective of respect for the human rights of users, the principle is to ensure that the social damage caused by the penalty does not exceed the damage to health caused by the drug.^15^

A major challenge that arises when considering the pros and cons of introducing penalties for cannabis use is the lack of full‐scale epidemiological studies on the health effects of cannabis use in Japan. Internationally, the safety of cannabis has been proven to be superior to that of alcohol, tobacco, and stimulants, according to a 2010 study by Nutt et al.^16^

Meanwhile, Matsumoto et al.^17^ conducted a small survey of 71 Japanese patients with cannabis‐related psychiatric disorders on the health effects of cannabis.

In their study, Matsumoto et al.^18^, ^19^, ^20^ identified long‐term cannabis use and the use of cannabis products with high THC content as risk factors for the development of cannabis use disorders, similar to the findings of previous international studies. In contrast, no significant association of cannabis‐induced chronic psychosis with the frequency of THC intake, duration of use, other psychiatric comorbidities, or genetic factors was noted. Moreover, those who had used concentrated cannabis products had a lower incidence of chronic psychosis. These results contradicted those of previous foreign studies.^21^

This study was conducted on patients undergoing psychiatric treatment, thus reflecting its specificity, and does not accurately reflect the reality of health disorders among cannabis users in general. However, to the best of our knowledge, this is the only study to have investigated the causes of cannabis‐induced health disorders (cannabis use disorders and cannabis psychosis) in Japan. Therefore, we conducted an online cross‐sectional survey on the health effects of cannabis among those who had used cannabis illegally in Japan from January to February 2021 and received 4138 valid responses.

The study defined cannabis health hazards as “cannabis use disorder (CUD),” “acute intoxication (AI),” “cannabis psychosis (CP),” and “amotivational syndrome (AS),” and determined the percentage of patients who were deemed eligible on the online checklist as the primary outcome. The rates of CUD were 8.3% and 9.5% among lifetime and current users, respectively. The AI rates were 38.5% and 41.5% among lifetime and current users, among whom 0.12% and 0.15% experienced severe symptoms requiring medical attention, respectively. Furthermore, 1.3% and 1.5% of lifetime and current users developed CP, while 2.7% and 2.9% developed AS, respectively.

Regarding social function, 94.7% of the respondents in this study answered that they are socially functioning, compared to 49.3% as reported in a previous survey conducted in Japan by Matsumoto et al.^17^ using the same questionnaire. This suggests that the health hazard from cannabis use in Japan is smaller than previously assumed.^22^

The current study aimed to identify the risk factors for health hazards associated with cannabis use by conducting additional analyses of correlations with background factors, such as genetic background, social life history, and actual cannabis use, among users who did and did not experience cannabis‐induced health hazards.

## 2 METHODS

### 2.1 Survey procedure

Data were collected via an anonymous, self‐administered questionnaire using Google Forms. The questionnaire URL was distributed via the social media platform of Green Zone Japan (blogs, YouTube, email newsletters, Twitter, and Instagram) between January 21 and February 3, 2021.

Green Zone Japan is a non‐profit organization dedicated to medical cannabis education and leading the discussion on the deregulation of medical cannabis in Japan.

This study was conducted by Green Zone Japan and the National Center of Neurology and Psychiatry (NCNP) and approved by the Ethical Review Committee of the NCNP (approval number: A2020‐117; date of approval: January 8, 2021). Furthermore, it was conducted according to the principles of the Declaration of Helsinki. The participants provided informed consent and anonymity has been preserved.

This study's inclusion criteria were as follows: (1) Japanese residence and nationality and (2) cannabis use at least once within the preceding year. The exclusion criteria were as follows: (1) disagreement with the content of the survey, (2) previous participation in the survey, (3) current overseas residence, and (4) non‐use of cannabis before or within the preceding year. Only responses that met these criteria were included in the analysis, which resulted in 3142 cases out of 4138 respondents.

### 2.2 Questionnaire

The online questionnaire collated responses to the following items in a multiple‐choice, open‐ended format: (1) demographic factors (sex, age, nationality, and place of residence); (2) social life history (educational background and employment status); (3) criminal history (criminal history of drug‐related and other crimes); (4) clinical genetic information (family history of mental illness, substance abuse/addictive behavior, and suicide attempts); (5) detail of cannabis use (age of initial use, recent use, psychiatric visits related to cannabis use, and the use of cannabis products other than dry flower); (6) use of other drugs; (7) mental health hazards related to cannabis use; (8) history of mental illness, self‐reported name of illness, and time of diagnosis; and (9) history of suicide‐related events (suicidal ideation and suicide attempts).

Four categories of cannabis‐related mental health hazards were discussed: CUD, AI, CP, and AS. The following criteria were applied to assign an individual to their appropriate category.

1. Cannabis use disorder

We used the diagnostic criteria for CUD provided in the Diagnostic and Statistical Manual of Mental Disorders, Fifth Edition, (DSM‐5) in our questionnaire. Those who answered “yes” to two or more of the following items were considered to have CUD.

A: Cannabis is often used in larger amounts or over a longer period than intended.

B: There is a persistent desire or unsuccessful efforts to reduce or control cannabis use.

C: Spending considerable time acquiring, using, or recovering from the use of cannabis.

D: Craving, strong desire, or urge to use cannabis.

E: Recurrent cannabis use resulting in failure to fulfill obligations at work, school, or home.

F: Continued cannabis use despite having persistent or recurrent social or interpersonal problems caused or exacerbated by the effects of cannabis.

G: Important social, occupational, or recreational activities are ceded or reduced owing to cannabis use.

H: Recurrent cannabis use in situations in which it is physically hazardous.

I: Continued cannabis use despite knowledge of the presence of a persistent or recurrent physical or psychological problem that is likely to have been caused or exacerbated by cannabis.

J: Developing extreme tolerance.

K: Appearance of withdrawal symptoms.

2 Acute intoxication

Participants who answered “yes” to the question “Have you ever had unwanted acute symptoms that could be attributed to cannabis?” in the questionnaire were classified as those with AI. Those who indicated that they required assistance from others and that their symptoms lasted >24 h were classified as “severe cases,” and those who indicated that they had CP (listed below) were excluded.

3 Cannabis psychosis

Participants who answered “yes” to the question “Have you ever experienced a chronic mental disorder triggered by cannabis use?” were considered to have CP.

4 Amotivational syndrome

Those who answered “yes” to the questions “Has the use of cannabis affected your work, that is, missing workdays, job loss, or decreased performance?” and “Since you started using marijuana, have you socialized less with others, gone out less often, or lost interest in your hobby activities?” were considered to have AS.

The questionnaire employed in this study is the same as the survey conducted by Matsumoto et al.^17^ as part of the Ministry of Health and Labor Grants Research Project and has been used in Japan.

### 2.3 Statistical analysis

Based on the collected data, participants were categorized into two groups based on CUD and/or CP development according to the DSM‐5. Binomial logistic regression analysis was subsequently conducted using CUD and CP as dependent variables to examine the factors associated with these two psychiatric disorders. The independent variables used in this study were similar to those used in a similar multivariate analysis by Matsumoto et al.^14^ The independent variables included the following: (1) family history of mental health problems (mental disorder, substance dependence/addictive behavior, and suicidal behavior), (2) mental disorder preceding cannabis use, (3) age of initial cannabis use, (4) duration (years) of cannabis use, (5) use of cannabis products other than dry flower, and (6) concurrent use of other psychoactive substances. These variables were selected, and forced entry was made in bivariate and multivariate analyses.

SPSS (version 26, IBM Corp., Armonk, NY) was used for all statistical analyses, with a significance level of 5%. Since all age‐confirmation items in the questionnaire were in the form of age categories, the highest age in the age category was used as the actual age (e.g., if a respondent marked the 19–24‐year age range, their actual age was assumed to be 24 years). The duration of cannabis use was calculated by subtracting the “age of initial cannabis use” from the “current age.”

## 3 RESULTS

Of the survey responses received, 3142 respondents who were Japanese citizens, resided in Japan, and had a history of cannabis use within the past year were included in the analysis. The profile of the participants is summarized in Table 1. With regard to sex, 2634 (83.8%) participants were men, while 508 (16.2%) were women, and their mean age was 31.1 years (±1 standard deviation [SD]: 9.6). Of the respondents, 2968 (94.4%) were socially functional.

**TABLE 1:** Respondent profile

Characteristic		*N* = 3142
Sex, *n* (%)	Male/female	2634 (83.8%)/508 (16.2%)
Age (years)	Mean (±1 SD)	31.1 (9.6)
Social function, *n* (%)	Functioning (working or schooling)/not functioning	2968 (94.4%)/174 (5.6%)
History of MI prior to cannabis use, *n* (%)	+/−	162 (5.2%)/2980 (94.8%)
Family history of MI, SUD, or SA, *n* (%)	+/−	801 (25.5%)/2341 (74.5%)
Age at cannabis‐use initiation (years)	Mean (±1 SD)	21.52 (4.34)
Duration of cannabis use (years)	Mean (±1 SD)	9.60 (9.33)
Experience of CODF, *n* (%)	+/−	2426 (77.2)/716 (22.8)
Experience of DOC, *n* (%)	+/−	3050 (97.1)/92 (2.9)
Cannabis use disorder, *n* (%)	+/−	300 (9.5)/2842 (90.5)
Cannabis psychosis, *n* (%)	+/−	46 (1.5)/3096 (98.5)

Abbreviations: CODF, cannabis products other than dry flower; DOC, drugs other than cannabis; MI, mental illness; SA, suicide attempts; SD, standard deviation; SUD, substance use disorder.

In total, 801 (25.5%) participants had a family history of mental health problems and 162 (5.2%) had a personal history of mental illness prior to cannabis use. The mean age of initial cannabis use was 21.5 years (±1 SD: 4.3) and the mean duration of use was 9.6 years (±1 SD: 9.33). A total of 2426 (77.2%) participants had used cannabis products other than dry flower, while 3050 (97.1%) had used drugs other than cannabis, such as alcohol. Of the respondents, 300 (9.5%) had CUD and 46 (1.5%) had CP.

Table 2 shows the results of the bivariate and multivariate analyses with CUD as the dependent variable. In the bivariate analysis, the factors significantly associated with a CUD diagnosis were “cannabis‐use initiation at a young age” (*p* < 0.001, OR: 0.942, 95% confidence interval [CI, 0.912–0.974]), “family history of mental health problems” (*p* < 0.001, OR: 2.017, 95% CI [1.575–2.583]), and “use of cannabis products other than dry flower” (*p* < 0.001, OR: 2.864, 95% CI [1.945–4.216]).

**TABLE 2:** Multivariate analysis of factors associated with cannabis use disorder

	Cannabis use disorder				Bivariate analysis					Multivariate analysis				
	+		−		*β*	*p*	Exp (*β*)	95% CI		*β*	*p*	Exp (*β*)	95% CI	
	*N* = 300		*N* = 2842					LL	UL				LL	UL
	Mean	SD	Mean	SD										
Age of first cannabis use (years)	20.68	3.55	21.60	4.41	ｰ0.060	<0.001	0.942	0.912	0.974	ｰ0.05	0.004	0.951	0.920	0.984
Duration of cannabis use (years)	10.53	9.17	9.50	9.34	0.011	0.069	1.011	0.999	1.024	0.007	0.261	1.007	0.994	1.021

	+	%	+	%										
Family history of mental health problems	117	39.0%	684	24.1%	0.702	<0.001	2.017	1.575	2.583	0.687	<0.001	1.988	1.545	2.556
Psychiatric disorders preceding cannabis use	22	7.3%	140	4.9%	0.424	0.075	1.527	0.958	2.434	0.513	0.039	1.671	1.026	2.722
Experience using cannabis products other than dry flower	270	90.0%	2156	75.9%	1.052	<0.001	2.864	1.945	4.216	1.002	<0.001	2.725	1.844	4.026
Concurrent use of other psychoactive substances	296	98.7%	2754	96.9%	0.861	0.095	2.365	0.862	6.487	0.784	0.131	2.190	0.791	6.062

Furthermore, multivariate analysis was conducted to circumvent the influence of confounding factors on the independent variables. “Family history of mental health problems” (*p* < 0.001, OR: 1.988, 95% CI [1.545–2.556]), “psychiatric illness preceding cannabis use” (*p* = 0.039, OR: 1.672, 95% CI [1.026–2.722]), and “use of cannabis products other than dry flower” (*p* < 0.001, OR: 2.725, 95% CI [1.844–4.026]) were identified as factors significantly associated with CUD diagnosis.

Table 3 shows the results of the bivariate and multivariate analyses with CP as the dependent variable. The bivariate analysis revealed that “cannabis‐use initiation at a young age” (*p* = 0.011, OR: 0.889, 95% CI [0.811–0.973]) and “family history of mental health problems” (*p* = 0.001, OR: 2.726, 95% CI [1.520–4.890]) were factors significantly associated with CP diagnosis.

**TABLE 3:** Multivariate analysis of factors associated with cannabis psychosis

	Cannabis psychosis				Bivariate analysis					Multivariate analysis				
	+		−		*β*	*p*	Exp (*β*)	95% CI		*β*	*p*	Exp (*β*)	95% CI	
	*N* = 46		*N* = 3096					LL	UL				LL	UL
	Mean	SD	Mean	SD										
Age of first cannabis use (years)	19.98	3.27	21.54	4.35	ｰ0.118	0.011	0.889	0.811	0.973	ｰ0.119	0.011	0.888	0.811	0.973
Duration of cannabis use (years)	11.41	8.74	9.57	9.34	0.019	0.186	1.019	0.991	1.048	0.017	0.257	1.017	0.987	1.048

	+	%	+	%										
Family history of mental health problems	22	47.8%	779	25.2%	1.003	0.001	2.726	1.520	4.890	0.928	0.002	2.531	1.400	4.576
Psychiatric disorders preceding cannabis use	5	10.9%	157	5.1%	0.825	0.086	2.283	0.890	5.857	0.972	0.052	2.644	0.990	7.051
Experience using cannabis products other than dry flower	34	73.9%	2392	77.3%	ｰ0.182	0.592	0.834	0.430	1.169	ｰ0.309	0.368	0.734	0.375	1.439
Concurrent use of other psychoactive substances	45	97.8%	3005	97.1%	0.309	0.761	1.363	0.186	9.995	0.299	0.770	1.348	0.182	9.995

In addition, multivariate analysis revealed that factors significantly associated with CP were “cannabis‐use initiation at a young age” (*p* = 0.011, OR: 0.888, 95% CI [0.811–0.973]) and “family history of mental health problems” (*p* = 0.002, OR: 2.531, 95% CI [1.400–4.576]).

## 4 DISCUSSION

To date, this study constitutes the largest cross‐sectional survey of cannabis users in Japan. The following is a discussion of the results obtained in this analysis.

### 4.1 Risk factors for CUD

In this study, four factors were identified in multivariate analysis as having an association with CUD: (1) “family history of mental health problems,” (2) “mental illness preceding cannabis use,” (3) “cannabis‐use initiation at a young age,” and (4) “history of use of cannabis products other than dry flower.”

Prior studies have identified the frequency and duration of cannabis exposure,^18^, ^19^, ^20^ use of THC‐rich products,^23^, ^24^ younger age at initiation of use,^25^ the presence of psychological distress^25^ (e.g., the prior onset of mental disorders) prior to initiation of use,^26^, ^27^ family history of substance use disorders and genetic influences,^23^, ^26^, ^27^ frequent arguments with partners and tendency toward violence,^28^ and other socio‐psychological factors as factors associated with cannabis‐related health problems. Therefore, with the exception of the duration of cannabis use, the results of this study corroborate those of previous studies.

The relationships between the four factors identified in this study and CUD were all cross‐sectional associations, and multiple hypotheses could be postulated for each causal relationship.

1. A family history of mental health problems might have facilitated the development of cannabis use disorders because of psychiatric vulnerability at the biological level. Nonetheless, the nurturing environment, characterized by mental health problems in the family, could have also been of influence. The identification of factors bearing a greater influence was not possible based on the data collected in this study.
2. The incidence of psychiatric disorders preceding cannabis use suggests that psychiatric disorders potentially promote CUD morbidity in later years. Alternatively, cannabis use might have served, at least temporarily, as a self‐medication for psychological distress caused by symptoms of mental disorders.^25^

However, it should be noted that a person who uses cannabis for medical purposes may use it habitually on a daily basis, thus meeting the diagnostic criteria for CUD in this study. In such people, having CUD does not necessarily have immediate morbid implications.

3 Regarding cannabis‐use initiation at a young age, drug dependence develops easily at a young age when the central nervous system is immature,^29^ and cannabis use during adolescence potentially bears the risk of pharmacologically promoted dependence. Notwithstanding, harsh social conditions have been reported to lead to the early initiation of cannabis use, and early bereavement of parents, adolescent delinquency, and negative mental states are reportedly related to CUD development.^23^, ^26^, ^30^, ^31^ Therefore, a harsh upbringing may possibly act as a confounding factor between the early initiation of cannabis use and CUD development.
4 Regarding the use of cannabis products other than dry flower, addicts might have also developed tolerance to THC, thus using concentrated products for their psychoactive effects, though exposure to high‐THC products, such as dabs, hashish, wax, and other THC concentrates, could have caused dependence.

In this study, the duration (years) of cannabis use was not significantly associated with CUD, as noted in previous studies.^18^, ^19^, ^20^ One reason for this observation may be the use of social networking services in this study, which potentially skewed the respondent population toward younger age groups. Resultantly, the sample size of users with a long history of cannabis use was not sufficient to reveal an association with the duration (years) of use (the average duration of cannabis use among the participants in this study was 10.8 years).

Collectively, it is reasonable to assume that several factors, including early initiation of cannabis use, use of products with a high THC content, genetic and constitutional factors, and comorbid psychiatric disorders, play a complex role in influencing susceptibility to CUD.

### 4.2 Risk factors for CP

In the present study, multivariate analysis identified (1) “family history of mental health problems” and (2) “cannabis‐use initiation at a young age” as risk factors for CP. Previous studies have identified family history of psychiatric disorders,^32^, ^33^ cannabis use early in life,^24^ use of products with a high THC content,^21^ and prior psychiatric illness^34^ as risk factors for CP development. In this study, the early initiation of cannabis use and a family history of mental health problems were consistent with the factors identified in previous studies.

A family history of mental health problems suggests that inborn and biological vulnerability to mental disorders may not be negligible.^35^ Gene mutations associated with CP, such as *AKT1* mutations, are also reportedly associated with the risk of developing schizophrenia.^35^, ^36^ Families harboring such gene mutations are potentially more vulnerable to the pharmacological effects of cannabis.

Regarding the initiation of cannabis use at a young age, the effects of THC exposure have been suggested to be potentially stronger at a young age, when the central nervous system is still developing, and may act in a pro‐psychotic manner.^37^

However, what was identified as CP in this study may actually have been schizophrenia that developed independently of cannabis use. This is because the most common differential diagnosis for CP is schizophrenia; nevertheless, the age of onset of cannabis use overlaps with the age of schizophrenia onset, and the participants in this study predominantly comprised young men.

In Japan, schizophrenia and CP are diagnosed in a mutually exclusive manner, and a single history of cannabis use tends to lead to a diagnosis of CP rather than schizophrenia. As stated in the first report of our study, the rate of CP among the participants was 1.3%.^22^ This figure is not significantly different from the lifetime prevalence of schizophrenia, which is about 1.0%.^38^ In clinical practice, a diagnosis of CP should be made after a thorough examination of the causal relationship between cannabis use and symptoms, rather than immediately jumping to the diagnosis of CP if there is a history of cannabis use.

Unlike previous studies, this study did not identify any association between a history of psychiatric disorders and CP, possibly because a tendency to diagnose even a single episode of cannabis use as CP rather than schizophrenia exists in Japan, and this perception appears to be widespread among the general population of cannabis users. Therefore, pre‐existing schizophrenia could have been misdiagnosed as a cannabis‐related disorder, resulting in the present results.

No association between the use of products with a high THC content and CP was observed. A similar study^17^ conducted in Japan using the same questionnaire in patients with CUD in psychiatric hospitals found that, unlike the findings of previous foreign studies, the use of products with a high THC content and other psychiatric comorbidities were negatively correlated with CP. The study assumed that many chronically psychotic patients who had used cannabis potentially had schizophrenia, and their immersive lifestyles might have isolated them from the cannabis‐using community and prevented them from receiving information regarding cannabis products other than dry flower.^17^ A similar possibility could have existed in the present study.

In this study, as in most previous studies, no significant association between the duration of cannabis use and CP was noted. Some previous studies have reported a dose–response relationship between CP and cannabis use^39^; however, in the United Kingdom, cannabis use has increased dramatically over the past 30 years, while the incidence of psychosis has not, and it has been estimated that to prevent a single case of CP, 2800–4700 people would need to stop using cannabis.^40^ In summary, genetic predisposition is a major contributor to CP development, and for those who are not predisposed, no simple dose–response relationship between cannabis exposure and CP exists.

### 4.3 Study limitations

This study has various limitations, six of which are primary. First, the diagnosis of CUD or CP was made via self‐administered questionnaire items and was not directly synonymous with the conventional clinical diagnosis. In particular, there is not a well‐developed consensus on the definition of CP, and there may be a mix of respondents with various medical conditions among CP respondents.

Second, the study was based on retrospective, self‐reported data collection, and the effects of recall bias cannot be excluded with respect to the frequency and duration of cannabis use. Third, the present study did not analyze detailed data on psychiatric disorders or the use of other psychoactive substances as controls. For example, patients with schizophrenia or bipolar disorder were likely to behave differently; nonetheless, this study analyzed them indiscriminately. Fourth, this study did not examine mental disorders, addictions/behavioral dependencies, and suicide attempts separately but analyzed them collectively as a “family history of mental problems.” Essentially, these events are considered to possess completely different biological and genetic backgrounds, and the impact of each event on the patient's upbringing may differ slightly. Fifth, the associations exhibited in this study are merely cross‐sectional and do not imply a causal relationship. To overcome this limitation, a large prospective cohort study is warranted. Finally, participant recruitment in this study was conducted via the Internet, and access to the survey was presumably generationally biased, posing a challenge in terms of the target population's representativeness. It is possible that there is a bias toward a higher estimate of cannabis safety since the conductor of this survey is an organization that disseminates information favorable to cannabis.

Despite the above limitations, our findings are generally consistent with those of previous studies, and it is highly probable that this study has a certain degree of validity. Above all, this study involved the largest sample size compared with previous related studies and is currently the only study in Japan to have targeted cannabis users in the community, rendering it of great academic significance.

## 5 CONCLUSIONS

We collected data on the social life backgrounds, cannabis‐use backgrounds, and rates of adverse events among the general population of cannabis users in Japan and conducted a multivariate analysis to identify the risk factors for major adverse events, namely, CUD and CP. The study identified the following as risk factors for CUD: (1) “family history of mental health problems,” (2) “psychiatric disorders preceding cannabis use,” (3) “cannabis‐use initiation at a young age,” and (4) “experience using cannabis products other than dry flower,” while the following risk factors for CP were identified: (1) “family history of mental health problems” and (2) “cannabis‐use initiation at a young age.” To the best of our knowledge, this study constitutes the first and only large‐scale survey of community cannabis users in Japan, and its results are consistent with those of previous studies, bearing a certain degree of validity.

## AUTHOR CONTRIBUTIONS

YM initiated the study and drafted the study design and the online survey together with YA and TM. TS collected the data and TM conducted the statistical analysis. YM and TM wrote the introduction, methods, results, discussion, and conclusion. All the authors have approved the final version of the manuscript.

## FUNDING INFORMATION

There are no funders to report for this submission.

## CONFLICT OF INTEREST

The authors declare no conflict of interest.

## ETHICS STATEMENT

This study was approved by the Ethical Review Committee of the National Center of Neurology and Psychiatry (approval number: A2020‐117; date of approval: January 8, 2021).

## INFORMED CONSENT

The participants provided informed consent and anonymity has been preserved.

## Supporting information


**Data S1:** Supporting information

## Data Availability

The data that supports the findings of this study are available in the supplementary material of this article.
